# What's the problem with patient experience feedback? A macro and micro understanding, based on findings from a three‐site UK qualitative study

**DOI:** 10.1111/hex.12829

**Published:** 2018-09-22

**Authors:** Laura Sheard, Rosemary Peacock, Claire Marsh, Rebecca Lawton

**Affiliations:** ^1^ Bradford Institute for Health Research Bradford Teaching Hospitals Bradford UK; ^2^ Bradford Institute for Health Research Bradford Teaching Hospitals and School of Psychology University of Leeds Leeds UK

**Keywords:** patient experience, patient feedback, qualitative research, quality improvement, United Kingdom

## Abstract

**Context:**

Collecting feedback from patients about their experiences of health care is an important activity. However, improvement based on this feedback rarely materializes. In this study, we focus on answering the question—“what is impeding the use of patient experience feedback?”

**Methods:**

We conducted a qualitative study in 2016 across three NHS hospital Trusts in the North of England. Focus groups were undertaken with ward‐based staff, and hospital managers were interviewed in‐depth (50 participants). We conducted a conceptual‐level analysis.

**Findings:**

On a macro level, we found that the intense focus on the collection of patient experience feedback has developed into its own self‐perpetuating industry with a significant allocation of resource, effort and time being expended on this task. This is often at the expense of pan‐organizational learning or improvements being made. On a micro level, ward staff struggled to interact with feedback due to its complexity with questions raised about the value, validity and timeliness of data sources.

**Conclusions:**

Macro and micro prohibiting factors come together in a perfect storm which provides a substantial impediment to improvements being made. Recommendations for policy change are put forward alongside recognition that high‐level organizational culture/systems are currently too sluggish to allow fruitful learning and action to occur from the feedback that patients give.

## 1 INTRODUCTION

The patient experience agenda is reaching a zeitgeist moment in many health‐care systems globally. Patients are increasingly giving feedback on their experiences of health care via a myriad of different methods and technologies. Most commonly, these take the form of national surveys, formal complaints and compliments and social media outlets. Various publications outline a range and diversity of qualitative methods for gaining rich feedback from patients.^1^ Several systematic reviews have identified a range of quantitative survey tools which are used across the world to capture patient experience in an inpatient setting.^2^, ^3^ These include large‐scale surveys such as the NHS National Inpatient Survey in the UK and the Hospital Consumer Assessment of Healthcare Providers and Systems in the United States.^3^ Currently, in the UK, major resource is being given to the collection of the Friends and Family Test^4^ which has been mandatory since 2014 for all acute hospital Trusts to collect.

A significant driving force for the current focus on gathering patient experience feedback in the UK arose from national level recommendations such as the Francis and Keogh reports.^5^, ^6^ Internationally, “Better Together” in the United States and “Partnering with Consumers” in Australia demonstrate that this focus has been mirrored internationally.^7^ It is now widely acknowledged that patients want to give feedback about health care^8^ and recommended that staff listen to what their patients say about the experience of being in hospital.^9^ Yet, whether staff can use this feedback to make changes to improve the experiences that patients have is now a central concern.^10^, ^11^, ^12^, ^13^, ^14^, ^15^ This pertains to differing areas of the health‐care system from senior management at the level of the hospital board (formalized group of directors) down to individual clinicians working on the frontline. Hospital boards have received recent attention to understand the ways in which they use patient feedback to improve care at a strategic level^16^ and how they govern for quality improvement.^17^ There is a concern that the ever growing collection of feedback is not being used for improvement but, rather, represents a “tick box mentality” of organizations thinking they are listening to their patients views but actually not doing so.^18^ Recent work in the UK has looked at how health‐care professionals make sense of why patients and families make complaints about elements of their care^19^ and found that it was rare for complaints to be used as grounds for making improvements.

Almost everyone interested in health‐care improvement, and certainly those providing frontline care now have a vested interest in listening to patients.^15^ However, a myriad of challenges are still preventing the wide‐scale effective *use* of patient feedback data for quality improvement. This can be contrasted against a backdrop of a simultaneous “movement for improvement”^20^ where grassroots, bottom‐up approaches to health‐care improvement are being championed. It is interesting to note that, despite the recent paradigm shift in the literature which acknowledges this “patient feedback chasm”,^21^ most commentators have so far only paid attention to the problems at the micro level.

Flott et al^11^ discuss problems related to data quality, interpretation and analytic complexity of feedback and then put forward ideas for how the data itself could be improved to allow staff to engage with it better. Likewise, Gleeson et al^12^ found a lack of expertise amongst staff to interpret feedback and issues surrounding the timeliness of it, coupled with a lack of time to act on the data received. Sheard et al^15^ explored why ward staff find it difficult to make changes based on patient feedback. They found that effective change largely relates to an individual or small teams structural legitimacy within the health‐care system and that high‐level systems often unintentionally hindered meso and macro level improvements which staff wished to make.

In this study, we report the findings from a qualitative study undertaken at three hospital Trusts in the North of England. We were interested in which types of patient experience data were being collected, how staff were or were not using this data and whether there was a relationship to improvement on the wards. Here, we base our reporting on the question “what is impeding the use of patient experience feedback?” which is examined through both a macro and micro lens. We concentrate on this finding as it arose from the participants as being of central importance. It is important to define what we mean by the use of the terms “macro” and “micro” within this study. Here, macro refers to the system, organization, structure or strategy, for instance, the hospital culture, how teams or processes are set up or ways of working. Micro refers to the issues with sources of feedback and how individuals use or interact with them.

## 2 METHOD

### 2.1 Setting

We conducted a mixed‐method qualitative study using focus groups and interviews across three NHS hospital Trusts in the North of England. This qualitative study was the first work package in a programme of research; whereby, the overall purpose was to develop a patient experience improvement toolkit to assist ward staff to make better use of patient experience feedback. The three Trusts were selected to provide diversity in size and patient population. Then, two wards per Trust were approached to take part in the study leading to six wards working with us. We sampled the six wards based on a divergence of speciality, size and patient throughput. The specialities of the wards were as follows: accident and emergency department, male surgery (this represents two wards at different Trusts), maternity department (including ante‐ and postnatal services), female general medicine and an intermediate care ward for older patients.

### 2.2 Sampling

Ward staff mostly represented opportunistic sampling, and management participants were sampled for maximum variation. Ward staff predominantly encompassed senior and junior nursing staff, support workers and the inclusion of allied health professionals in some of the focus groups. Management participants were drawn from a range of roles occupying middle‐ and senior‐level hospital management such as patient experience managers or heads of patient experience, matrons, heads of nursing (and their deputies), research leads, medical, quality, risk, governance and performance directors. The bulk of interview participants worked directly in or managed patient experience teams.

### 2.3 Data collection

Fieldwork took place between February and August 2016. University of Leeds ethical approval was secured in October 2015 (ref: 15‐0258), and NHS Health Research Authority governance approval was granted in February 2016. All participants gave written, informed consent. Ward staff took part in focus groups, and management staff took part in individual in‐depth interviews. Seven focus groups and 23 individual interviews were conducted. Focus groups ranged from three to seven participants, and two management participants were interviewed as a dyad. The average length of an interview was 55 minutes and 45 minutes for a focus group. In total, 50 participants took part in this qualitative study. All focus groups and interviews were conducted face to face in staff offices, digitally recorded and then transcribed by a professional transcriber. RP collected all interview data. LS, RP and CM all collected focus group data. All are experienced qualitative health researchers with doctorates in their respective fields.

### 2.4 Topic guide questions

Two topic guides were devised; one for the data collection from ward staff and another for management participants. Headline topic guide questioning was derived from the literature.^1^, ^2^, ^3^, ^9^, ^14^ Focus group questioning centred on what types of patient experience feedback the participants received, how they engaged with it and responded to it and where/how it fitted in with their everyday clinical work. Interview questioning explored the different kinds of patient experience feedback available to the Trust and how these were generated, prioritized and managed at the level of the ward, directorate and whole organization. The formats of the topic guides and that of interview questioning were flexible to allow participants to voice what they considered to be important. Both topic guides were piloted, with changes being made to the content and structure based on how initial participants responded to the interview or focus group questions. We included all data in the analysis and did not discard meaningful data which were gathered during piloting.

### 2.5 Analysis

LS and RP took the same five interview transcripts, and each independently developed a provisional descriptive coding framework. These five transcripts were chosen as those which were representative of the whole interview data set in terms of spread across the Trusts and general content. The same exercise was repeated for the focus groups, albeit with three transcripts. LS and RP held an intense analysis session where they met (along with RL) to discuss the differences and similarities in their coding frameworks, although there was general parity amongst them. LS then returned to the selected transcripts and immersed herself in the data in order to devise an overall meta coding framework which would allow for data from both the interviews and focus groups to be coded together. This meta coding framework sought out themes on a conceptual level rather than a descriptive level. That is, rather than simply describing what the participants discussed, LS looked for the differing ways in which patient experience feedback was approached conceptually across the participants involved in both methods. Differences and similarities were identified with LS noticing that participants discussed the topic at different levels with the management interviewees tending to view patient experience feedback in a macro way (both explicitly and implicitly) and the ward staff focus group participants viewing it in a micro manner. The meta level coding framework was checked with RP for representativeness and accuracy. After slight modification, LS then coded all transcripts and some subthemes were modified as coding progressed. LS conducted further interpretive work to write‐up the findings. Initially, we began by conducting a classic thematic analysis^22^ but realized that this was not sufficient for our needs as thematic analysis often relies on portraying a descriptive account of participants’ narratives. Instead, we looked to generate high‐level conceptualizations from the data. The analysis was wholly inductive, and, as such, we did not structure it on any existing theoretical frameworks.

## 3 FINDINGS

Here, we briefly set the scene by describing the main sources of patient experience feedback in the UK before moving on to focus entirely on: “what is impeding the effective use of patient experience feedback?” All participants have been ascribed a number and a generalized descriptor of their role, rather than their precise role, to protect their identity. We will discuss two distinct groups of participants which we will call “ward staff” and “managers.”

### 3.1 Setting the scene**—**what are the sources of patient experience feedback?

All participants were able to name a wide variety of the types of patient experience feedback they had encountered and interacted with, in their professional roles. This took the form of formalized written sources such as the Friends and Family Test, complaints and compliments, thank you cards, Patient Advice and Liaison Service (PALS) communication, patient stories, NHS Inpatient Survey, local surveys and other initiatives such as “You Said, We Did.” Senior leaders within the organizations spoke about Care Quality Commission inspections and the use of social media as outlets for feedback, although ward staff paid less attention to these sources of data. (See Glossary for an explanation of specific sources of feedback). When first asked to discuss patient experience feedback, ward staff spoke about the more immediate, direct “in the moment” verbal feedback from patients on their ward which they received in an impromptu manner during the course of a shift. This often took the form of patients complaining verbally**—**in an informal manner**—**about their care or the environment to the clinician caring for them or to a more senior staff member. Conversely, it also included spontaneous thanks or praise given in an interpersonal exchange. In this study, we focus on formalized sources of patient experience feedback and discuss factors surrounding their effective use, as per our key areas of interest and research brief. However, it should be acknowledged that informal feedback was often used by ward staff in a timely way to improve the experience for the needs of a particular patient.

### 3.2 What is impeding the effective use of patient experience feedback?

We chose to focus on the factors that are impeding the use of feedback rather than an account which paid equal attention to the factors that were assisting. Whilst there were certainly instances where individual personnel and small teams had instigated processes and ways of working which were beneficial, these accounts were localized and not of sufficient importance to most participants about the topic at hand. Furthermore, attempts to improve issues identified in feedback sometimes led to unintended consequences which further problematized an already complex and fraught task. When participants talked in positive terms about patient experience feedback, they often spoke of idealized situations or what they would like to see happen in the future rather than what was currently happening in practice. Overwhelmingly, the participants interviewed across the data set pinpointed significantly more negative factors within their current working practices when trying to use patient feedback than positive and this is therefore where we place our analytic attention.

There is a clear division between a macro and micro understanding of how participants discussed patient experience feedback within their health‐care organization. Management participants commented on feedback and the use (or not) it serves at the level of the organization, whereas both ward staff and management pinpointed the problems at a micro level with the function and usefulness of the individual data collection sources.

#### 3.2.1 At the macro level of the health‐care organization

Considering the data set as a whole, possibly the most striking element is the overwhelming nature of the *industry* of patient experience feedback. Ward staff at one hospital department at Trust C stated they were collecting around a thousand FFT cards a month, in addition to all the other patient feedbacks received. Both management and some ward staff participants across the whole sample reported feeling overwhelmed and fatigued by the volume and variety of data that the Trust collected:

So we have got the Friends and Family Test, which produces, as I am sure that you are aware, reams and reams of information but nobody is really quite sure what to do with that information. Because there's just loads of it. I mean our goal is about 50% of people that leave fill in a card. (Trust B, Interviewee 2, Patient experience management)

At each of the three hospital sites, a significant, system‐wide level of resource, effort and time was being expended which primarily focused on maintaining the collection rates of feedback. This was coupled with layers of hierarchies and bureaucratic processes surrounding data collection which were said to be to be confusing to staff and patients alike. Mirroring the current NHS staffing situation amongst the clinical workforce, some management participants stated they did not have enough staff or appropriate expertise (often stated as qualitative expertise) in their immediate teams to be able to work effectively to produce meaningful conclusions from the data they received. This was despite an abundance of resource given over to collecting feedback on the ground, leading to a bizarre situation whereby masses of data were being collected from patients, but a lack of skill and personpower, within the patient experience team, prohibited its interpretation and therefore its use:

So with all the ways and means of collecting the feedback, it's how to actually pull out a theme to actually make an improvement. It feels as if we are overwhelmed with everything and the next step for me is, we need to actually take it to the next level and start learning from it. (Trust B, Interviewee 3, Patient experience management)

At the centre of this situation was the idea that data collection *in and of itself* was considered the most important achievement rather than a focus on how the feedback could be used to drive improvement. In relation to FFT, there was a narrow focus on each ward's response rate (what percentage of their patients had completed an FFT form) and enlarging this response rate at a detriment to other activities. Regarding complaints, there was an overt focus on both the timeliness of response to complaints and on trying to reduce the volume of them rather than an understanding of what an effective response looked like and how this could be emulated:

Number of complaints is one thing, great, are they [complaints team] getting more or less? Less, great. Are they responding to them within our forty day timescale? Yeah, great. For me that's all nice and boxes we can count and tick, but actually what are the main complaints? What are the main themes? What are they doing about them? What's on their action plan? So that we're, we want to shift to a more action based approach rather than counting. (Trust C, Interviewee 4, Performance manager).

Management participants often talked in corporate terms about where the responsibility for patient experience feedback sat within the hospital hierarchy, which demonstrated that patient experience was a fractured domain, spread across several different disciplines. However, some senior leaders articulated the artificial nature of this division and how this splintering of the response to patient feedback was hindering the ability for change to occur as a result of it. For instance, in one Trust the responsibility for complaints, PALS and FFT was split across three different teams who had little crossover and therefore minimal capacity to consider this wealth of feedback from patients as a whole. In a different Trust, a senior manager had noticed this division was holding learning back and brought representatives from these teams together once a month in a formal event. A few participants noted how the electronic systems for collating the different sorts of feedback were completely distinct, which further compounded the lack of cross‐team working. The division between complaints and PALS**—**both as a concept and practically**—**was remarked upon by some management participants as being arbitrary and unnecessarily confusing to patients and the public. Some participants spoke about how several different initiatives on patient experience were simultaneously ongoing within the same Trust, with little ability for the linkage between these to be made explicit as their remit was under different teams.

The participants interviewed for this study nearly all saw an immense value in patient experience feedback, and most believed it should receive a high priority at a strategic and Trust board level. Yet, this was not often the situation “on the ground” in their organizations and the culture around this was said to be hard to change. Patient experience was sometimes said to be the poor relation of patient safety and finance with a lesser emphasis and priority placed on it:

They [directorate representatives] have to give an explanation as to why performance is bad in terms of finance, access, targets, the waiting lists and quality is one of the agenda items, but it seems it will always be the item that is skimmed over. Patients experience and stuff, it is on there but no one ever really pays attention. (Trust C, Interviewee 3, Patient experience management)

Related to the above, management participants discussed where the responsibility for patient experience “sat” within their Trust. Usually, patient experience was housed under the nursing remit and patient safety under the medical remit. This division was said to be unhelpful by several participants who felt that patient experience was therefore automatically seen as an issue for corporate and shop floor nursing staff to solve:

My only nervousness is it's done almost entirely through nursing…and there's rafts of things [feedback] that are about doctors…I think there is a perception, you know, the doctors do the doctoring thing and nurses do the patient care thing and it's nurses and it's about wards when actually when you look at it, actually quite a large volume [of feedback] is nothing to do with nurses whatsoever. (Trust C, Interviewee 4, Performance manager)

In a drawing together of the points raised so far, it is clear that current patient feedback systems do not generally allow for learning across the organization. The collection of patient experience feedback seems to be the focal point, with an intensive resource given over to this, whilst fractured and disparate teams struggled to make sense of the data or to be able to assist ward staff to do so.

#### 3.2.2 At the micro level of the feedback itself

Both management and ward staff participants spoke about the usefulness of the patient experience feedback they received. Usefulness was often aligned to whether it was appreciated that improvements could be made based on the feedback. Overall, it was reported that most wards were awash with generic and bland positive feedback which rarely guided them in identifying specific elements of positive practice. This contrasted with a smaller amount of negative feedback where patients often pinpointed precise instances of poor patient experience:

Senior nurse: Usually the positives are very general, when they're negative it's something very specific; “the bins are noisy, the buzzers don't get answered on time, I didn't get X, Y and Z at teatime…”

Ward clerk: “My lunch was cold”

Senior nurse: Yeah, usually they're quite specific, whereas the good and the positives tend to be more general; “the whole ward was clean and tidy, the staff are all lovely” do you know what I mean? So I feel sometimes we don't always necessarily get that much information about the positives, it's always a very general positive. (Trust A, Focus group 2)

A different problem with the feedback sources currently received related to what extent ward staff were or were not able to interact with and interrogate the raw data which were passed onto them by patient experience team members. Senior ward staff participants were sent spreadsheets of unfiltered and unanalysed feedback. In some instances, this ran into hundreds of rows of text for a month's worth of data. The complexity and volume of the data that ward staff had to contend with were often seen as overwhelming to the extent that some ward staff deliberately chose not to engage with the data. The two main issues which prevented ward staff from using**—**or some cases even looking**—**at patient experience feedback were a lack of time and a lack of training. Taking time away from clinical duties to “sift through,” a large amount of unsorted data was not perceived to be a high priority. Likewise, it was evident that ward staff did not have the required skills to be able to perform sophisticated analytic tasks on the data they received.

The stark reality is most frontline staff, and even most managers, really struggle to find the time to look at the kind of in‐depth reporting we get back. We get reports back that are, you know, extremely bulky documents and people struggle to have the time to really read them, understand them, and use them. (Trust B, Interviewee 4, Patient experience management)

In general, the raw data from patients were said to be difficult for ward staff to interact with and some participants questioned whether the current process was fit for purpose. A few management participants spoke about how a lack of decent analysis before the data were passed onto ward staff simply worked to compound the problem even further. Even more difficult to achieve was the idealized notion that differing data sets should be brought together to provide an overall picture of what patients thought about an individual ward. Despite all of the above difficulties, there was an expectation by senior leaders that ward staff should be using the feedback to make improvements to the ward.

Compounding the above problems of data interrogation, were underlying problems that ward staff perceived to be inherent in the data already collected and therefore its value even before it reached them. Most significantly, timeliness was seen as one of the main concerns with it being difficult to engage ward staff with data that are not real time. A specific example of this is the NHS Inpatient Survey where patient feedback is viewed months after it has been collected. Frustrations were attached to receiving feedback which was considered historical if ward staff had already started to work on improvements to address known problems. Even FFT data were said to be too late if it reached ward staff a few months after it was collected. Ward staff participants struggled to remember the circumstances of a complaint if the complaint was made several months after the patient had stayed on the ward.

A specific idea raised by ward staff participants were the limitations of current patient experience feedback sources, particularly those that are nationally mandated such as FFT. Throughout the data set, there were numerous accounts of how FFT was considered “more bother than it was worth.” superficial, unhelpful and distracting. It was unfortunate to learn that in two Trusts, FFT had replaced several local patient feedback initiatives which ward staff had previously placed a large emphasis on as being of use to their everyday practice and learning:

Senior sister: I know the feedback we get from it [FFT] is not as good as what the You Said We Did information that we used to get back.

Senior midwife: Cos that was very, very specific wasn't it?

Senior sister: Yeah, it was, you could relate to it and you could look at it and you could help to action things. (Trust B, Focus group 1)

Considering the above micro view of the participants’ narratives, it can be seen that a large amount of feedback is positive but simultaneously generic in nature. Ward staff struggle to interact with how the feedback is presented to them in its current format, and there are questions raised over the inherent value of the sources, specifically in relation to factors such as timeliness.

## 4 DISCUSSION

From the findings given above, we can see how the ability for effective use to be made of patient experience feedback is hindered at both the micro level (of how individual clinicians and teams of staff have difficulty engaging with the data sources) and the macro level (how organizational structures are unwittingly preventing progress). This is played out through various means in a macro sense such as a lack of pan‐organizational learning, the intense focus on the collection of data at the expense of understanding how it could be used and fractured patient experience teams who want to assist ward staff but find this difficult. In a micro sense, a large amount of generic positive feedback is seen as unhelpful with ward staff struggling to interpret various formats of feedback whilst they question the value of it due to factors such as the timeliness and validity of the data. The macro and micro prohibiting factors come together in a perfect storm which provides a substantial impediment to improvements being made. The current study is the first to identify which concrete macro issues at the level of the organization are obstructing patient experience feedback being acted upon.

A meta principle that can be drawn from the findings of this study is that organizational culture in health care is not changing as fast as actors on the ground strive for it to change. For instance, there is already a recognition that too much data are being collected from patients in relation to the little amount of action that is taken as a result of it.^10^, ^18^ Our participants**—**particularly the management participants**—**were very mindful of this but largely seemed powerless to prevent the tsunami of ongoing data collection within their organization. Equally, it has been known about for some time that many members of ward staff find interpretation of data sets difficult or impossible as they have minimal or no training in analytics or quality improvement.^18^ This issue was raised by both management and ward staff participants in our study, but there was no strategy in place or forthcoming at any of the three organizations we studied to address this issue. The slow movement of culture change discussed above is likely to be related to what has recently been dubbed the “uber‐complexity” of health care,^23^ with key actors working within a system which favours centralized power structures over localized individualistic solutions.

### 4.1 Recommendations for change

There should be an organizational emphasis placed on the principle that all feedback collected ideally needs to have the ability be *meaningfully used* by those providing frontline care. Otherwise, it becomes unethical to ask patients to provide feedback which will never be taken into account. An immediate concentration on quality over quantity is important with a strategic focus which takes the priority off the collection of data and onto its use. Senior health‐care leaders, such as Trust directors, may need to lobby government to achieve this, particularly around the Friends and Family Test which is currently mandatory for Trusts to collect. Secondly, ward staff need to *understand* the formalized sources of feedback they are receiving from their patients before they can begin to use them. There are two approaches here, possibly complimentary, but both are difficult to achieve within the current NHS climate. One is that significant work needs to be undertaken upstream by patient experience teams to relay the data to ward staff in an accessible, straightforward and engaging manner. Another would be for a proportion of ward staff to be given robust training in how to understand and act on the feedback they receive from their patients. This should encompass analytic techniques and quality improvement methodologies. One without the other may prove ineffective and only allows staff to see half the picture. The macro influences the micro here because if patient experience teams were not overwhelmed by a volume and multiplicity of data sources whilst simultaneously underprovided with analytic resource, then this could potentially be accomplished.

At the level of the organizational structure, teams which have been tasked with a narrow focus on individual sources of data (eg, FFT, PALS and complaints) should be merged together with a strategic emphasis placed on learning across the organization from the variety of feedback sources. This does not necessarily require extra resource but a firm commitment to different ways of working which aim to understand the big picture instead of paying attention to the treadmill of targets and metrics per individual data source. If patient experience feedback is to be valued, then it should stop being viewed as the poor relation to patient safety and finance whilst simultaneously**—**and concertedly**—**moved outside the remit of being badged as a problem for corporate and shop floor nursing to solve.

### 4.2 Strengths and limitations

To our knowledge, this is the first paper which has paid significant attention to the system‐level, macro factors that are inhibiting the use of patient experience feedback. Other commentators^10^, ^11^, ^12^, ^18^ have noted some of the micro level factors we have identified here but not how they interact with structural issues problems which further compound the issue at hand. A limitation may be our explicit focus on the *problems* surrounding the use of patient experience feedback due to the emphasis that participants themselves placed on this aspect. It could be that a write‐up—which sought to pay equal attention to problems and solutions**—**may have uncovered different or more worthwhile suggestions for change.

## 5 CONCLUSION

Our study found that the use of patient experience feedback is impeded by issues which pertain to both macro level structural/organizational factors and micro level factors surrounding how individuals interact with the data sources. These factors collide to create a situation where an ever increasing amount and diversity of feedback is being collected, but simultaneously staff at different levels in the hospital hierarchy are struggling to use it to make improvements to patient care. Given the current movement towards the importance of paying attention to patient experience, it is likely that organizational culture and systems are moving too slow in response to how staff say they want to use patient feedback. We put forward recommendations for change which focus on quality over quantity, working towards ensuring ward staff can understand the data they are receiving and changes to organizational structure.

## CONFLICT OF INTEREST

The authors declare no conflict of interest.
